# Docetaxel in Non-Small Cell Lung Cancer: A Multi-Centre Real-World Evidence Analysis in the Immunotherapy Era

**DOI:** 10.3390/curroncol33050277

**Published:** 2026-05-08

**Authors:** Christopher F. Theriau, Yuchen Li, Deborah Akurang, Sara M. Moore, Rosalyn A. Juergens, Natasha B. Leighl, Paul Wheatley-Price

**Affiliations:** 1 Department of Medical Oncology, The Ottawa Hospital Cancer Centre, University of Ottawa, Ottawa, ON K1H 1C4, Canada; ctheriau@toh.ca (C.F.T.);; 2 Department of Medical Oncology, Cancer Centre of Southeastern Ontario, Queens University, Kingston, ON K7L 5P9, Canada; 3 Ottawa Hospital Research Institute (OHRI), Ottawa, ON K1Y 4E9, Canada; 4 Department of Medical Oncology, Juravinski Cancer Centre, McMaster University, Hamilton, ON L8V 5C2, Canada; 5 Department of Medical Oncology, Princess Margaret Cancer Centre, University of Toronto, Toronto, ON M5G 2M9, Canada

**Keywords:** docetaxel, immunotherapy, KRAS, real-world evidence, NSCLC

## Abstract

Docetaxel has been a standard second-line or later (2L+) treatment for advanced non-small cell lung cancer (NSCLC) for more than two decades and remains recommended in current treatment algorithms. However, real-world outcomes in the era of immune checkpoint inhibitors (ICI) are limited. This multicenter retrospective study included patients with advanced NSCLC treated with 2L+ docetaxel monotherapy between January 2011 and December 2020. The primary endpoint was median overall survival (mOS) from docetaxel initiation. Subgroup analyses were conducted to identify predictors of OS. A total of 285 patients were analyzed. Median age was 62 years; 43% female, 75% ECOG performance status (PS) 0–1, and 65% adenocarcinoma. Molecular alterations included EGFR (20%) and KRAS (15%). PD-L1 status was available in 66% of patients. Median docetaxel exposure was three cycles, administered as 2L (48%), 3L (35%), or 4L+ (17%). Prior therapies included chemotherapy (96%), ICI (38%), targeted therapy (21%), and chemo-immunotherapy (10%). mOS was 6.5 months (95% CI, 5.9–7.3). On multivariate analysis, KRAS alteration (HR 0.59; 95% CI 0.37–0.94; *p* = 0.026) and ECOG PS (1 vs. 0 HR 2.26; 95% CI 1.15–4.43; *p* = 0.018, ≥2 vs. 0 HR 2.62; 95% CI 1.27–5.41; *p* = 0.0091) remained independent predictors of OS. Real-world OS with docetaxel is consistent with historical trial data, irrespective of prior ICI, targeted therapy, or chemo-immunotherapy. KRAS mutation and a favourable ECOG PS were associated with improved survival.

## Simple Summary

Docetaxel remains a commonly used second-line or beyond treatment following progression on platinum-based chemotherapy, immunotherapy, and targeted agents, despite advances in checkpoint inhibitors and targeted therapies. Real-world evidence on its efficacy after modern first-line treatments remains limited. This study demonstrates that overall survival (OS) with second-line or beyond docetaxel in real-world settings is comparable to historical trial outcomes in patients previously treated with immunotherapy, chemoimmunotherapy, or targeted therapy. Predictive factors independently associated with improved OS included the presence of KRAS mutations in tumours and good performance status (ECOG 0–1). These findings support the continued use of docetaxel in the ≥second-line setting regardless of prior treatment modality, while emphasizing greater benefit in patients with favourable baseline characteristics. Conversely, patients with poor performance status (ECOG ≥ 2) experience increased hazard of death and should discuss the risk and benefit of ≥second-line docetaxel, with consideration given to alternative approaches, including best supportive care.

## 1. Introduction

Lung cancer remains one of the most common cancers and is the leading cause of cancer-related death worldwide, with non-small cell lung cancer (NSCLC) being the most common subtype [1]. Approximately 50% of all NSCLC are diagnosed at stage 4, while patients diagnosed at an earlier stage have high relapse rates, translating to an overall 5-year survival of 22% [2,3]. The therapeutic landscape of advanced stage NSCLC has changed significantly over the past two decades with a key treatment transition bringing immunotherapy and small-molecule targeted inhibitors to the first-line setting [4,5]. A subset of these patients, with a better performance status and who were previously treated with platinum-based regimens, may be treated with docetaxel monotherapy as a second-line or beyond of treatment.

Docetaxel was first adapted by the United States of America Food and Drug Administration (FDA) in 2000 for second-line (2L) treatment of NSCLC based on prolonged overall survival in two phase III trials, TAX 317 and TAX 320 [6,7]. Second-line docetaxel post platinum-based chemotherapy was initially shown to provide a median overall survival (mOS) of 7.0 months, compared to 4.6 months, without a significant increase in grade 3 or 4 non-hematologic toxicity [7]. To date, very few real-world studies have explored the efficacy of 2L+ chemotherapy after progression following 1L therapy, specifically now with the advent of targeted and immunotherapy. Many agents have been compared head-to-head against docetaxel after progression on platinum-based chemotherapy, ICI or CIO. Several chemotherapy agents have shown no OS benefit compared to docetaxel such as pemetrexed (adenocarcinoma) [8], topotecan [9] and vinorelbine/ifosfamide [6]. Notable tyrosine kinase inhibitors such as sotorasib (KRAS G12C) [10], gefitinib [11], and erlotinib [12], and antibody–drug conjugates such as datopotamab-deruxtecan [13] and sacituzumab-govitecan [14] have failed to show an OS benefit compared to 2L docetaxel. A few agents shown improved OS compared to docetaxel, but these are primarily ICIs that have been moved to 1L settings, including pembrolizumab [15], nivolumab [16] and atezolizumab [17]. Results with docetaxel monotherapy across previous trials have shown a mOS range of 6.4–11.3 months; although, differences existed between trials including inclusion of Eastern Cooperative Oncology Group (ECOG) 2 patients and prior therapies used [6,8,9,12,16,17,18,19,20,21,22,23].

Despite the advent of immune checkpoint inhibitors and targeted therapies, docetaxel remains a commonly used option after exhaustion of platinum-based chemotherapy, immunotherapy, and targeted agents. A recent retrospective study evaluating second-line chemotherapy after progression on first-line chemo-immunotherapy (CIO) demonstrated that patients with primary resistance (first-line PFS < 6 months) had significantly shorter second-line OS and PFS compared with responders [19].

There are few studies examining docetaxel in real-world settings after ICIs [24]. Based on previous trials, single-agent docetaxel has demonstrated modest clinical activity and significant hematologic toxicity. Thus, appropriate patient selection is critical. Currently, there is limited data available on potential clinical predictors of response to docetaxel. Therefore, our primary objective was to examine real-world responses to docetaxel chemotherapy as monotherapy in any post first-line of treatment for NSCLC and to assess for predictors of favourable response to docetaxel chemotherapy in patients treated with previous chemotherapy, chemo-immunotherapy, immunotherapy, or targeted therapy.

## 2. Materials and Methods

### 2.1. Study Design and Patients

Research ethics approval was granted for this study at each participating centre. In this retrospective multicentre cohort study, patients from 3 major cancer centres in Ontario, Canada (Princess Margaret Cancer Centre, Toronto, ON; Juravinski Cancer Centre, Hamilton, ON; The Ottawa Hospital Cancer Centre, Ottawa, ON) were included. We included patients aged ≥18 years who received docetaxel as any post-1L treatment (chemotherapy, chemo-immunotherapy, immunotherapy, targeted therapy) for advanced NSCLC over a 10-year period between 1 January 2011–31 December 2020. Inclusion criteria included histologically confirmed NSCLC, treatment with docetaxel monotherapy in second-line or beyond (2L+), stage IIIB/IV at time of starting docetaxel. The standard docetaxel treatment schedule was every 3 weeks. No weekly docetaxel regimen was used. Exclusion criteria included patients previously treated with docetaxel for any alternative malignancy, patients with concurrent diagnosis of another metastatic cancer, patients with missing treatment variables (treatment regimen, start/stop dates, number of cycles) prior to docetaxel and patients treated in a clinical trial if it could not be established that they received docetaxel monotherapy. Where available, molecular testing was reported for EGFR, ALK, ROS1, BRAF, KRAS and HER2. Due to our study period, early cases only reported EGFR and ALK testing primarily with polymerase chain reaction (PCR)-based methods and immunohistochemistry (IHC), respectively, with NGS testing occurring on patient samples later in our study period. ROS1, BRAF, KRAS and HER2 were not routinely tested unless necessary for clinical trial enrollment, with more standard NGS practices occurring later in our study period. Similarly, PD-L1 testing was reported where available, but was not performed in the earlier years of the period studied. Data was extracted from medical records until a database lock date of 21 November 2024.

### 2.2. Outcome Measures

The primary endpoint of this study was mOS in patients who received docetaxel as post-1L of therapy, defined as the date from first docetaxel treatment to date of death from any cause or last follow up with a data end point of 21 November 2024. Key secondary endpoints were mOS in cohorts who had or had not received any prior immunotherapy (ICI), and in cohorts with or without actionable genomic alterations. Other secondary endpoints included 2L+ docetaxel objective response rate (ORR) and disease control rate (DCR). ORR (complete response: CR + partial response: PR) and DCR (CR + PR + stable disease: SD) were investigator-assessed based on a retrospective review of radiology reports, imaging findings, and treating clinician documentation as recorded during routine clinical practice at each participating centre. Patients who died before undergoing first restaging imaging after initiation of second-line or later docetaxel were classified as death prior to response assessment (DPRA). We also used descriptive analysis of cohorts to determine treatment patterns and predictors of response for mOS differences to 2L+ docetaxel therapy for tumour histology, ECOG performance status (PS), previous curative intent therapy (surgical, radiation, chemotherapy, immunotherapy), sites of metastasis prior to docetaxel, 1L therapy used (platinum-based chemotherapy, immuno-mono/dual therapy, platinum-based chemoimmunotherapy, targeted therapy), line of palliative docetaxel use (2L, 3L, 4L+), PD-L1 status (≥50%, 1–49%, <1%), duration of previous line of therapy (≤6 months, >6 months) and prior chemotherapy regimen.

### 2.3. Statistical Analysis

Median values (interquartile range) and frequencies (percentage) were provided for descriptions of continuous and categorical variables, respectively. OS was described using median values with their 95% confidence intervals (95% CI). Product limit survival estimates were utilized to create survival probability with logrank test performed. Hazard ratios (HRs) for overall survival were estimated using Cox proportional hazards regression. Variables with *p* ≤ 0.05 in univariate analysis were included in the multivariable model. Proportional hazards assumptions were tested using Schoenfeld residuals. Analyses were conducted using SAS v9.4 (SAS Institute Inc., Cary, NC, USA). A *p*-value *<* 0.05 was considered statistically significant and all tests were two-sided.

## 3. Results

### 3.1. Characteristics of the Study Population (Table 1)

A total of 285 patients were included in the analysis. The median age was 62 years, 42.8% were female and 67.6% were either current (21.3%) or former smokers (46.3%). Most patients had an ECOG PS of 1 (58.5%) at the start of docetaxel treatment and had an adenocarcinoma histology (64.9%). Other histologies included 1.3% sarcomatoid carcinoma and 0.8% poorly differentiated carcinoma/basaloid SCC. PD-L1 status was available in 66% of patients and among those with available PD-L1 status the rates were 19.6% ≥50%, 25.9% 1–49%, 54.5% <1%. Of the 96 patients with missing PD-L1 testing, 46 were adenocarcinoma (24.8% of all adenocarcinomas) and 44 squamous cell carcinoma (56.4% of all SCCs). Previously, 25.9% of patients had been treated with curative intent treatment and all patients were stage IV at the start of 2L+ palliative docetaxel. Genetic testing was not available for 50 patients (17.5%) with seven being adenocarcinoma (3.8% of all adenocarcinomas), 40 SCC (51.3% of all SCCs), two large cell and one neuroendocrine. All tumour mutation rates were reported in a percentage based on those patients who had been evaluated for the desired actionable alteration. The most common molecular alterations were EGFR mutation (20.4%) and KRAS (15.4%) with mutation status available in 81% and 64%, respectively. Further analysis of KRAS mutation subtypes were KRAS G12A (46.4%), G12C (25.0%), G12V (14.3%), G12D (7.1%), G12S (3.5%) and G13D (3.5%).

A total of 274 (96.1%) patients were treated with palliative chemotherapy prior to 2L+ docetaxel, of which 202 (70.9%) and five (1.8%) were treated with palliative platinum-based or taxane (paclitaxel) chemotherapy, respectively. A total of 135 (47.4%) were treated with any prior ICI, of which 108 (38.0%) received either ICI monotherapy (PD-1 inhibitor: pembrolizumab, nivolumab or PD-L1 inhibitor: durvalumab, atezolizumab) or dual ICI therapy (durvalumab/tremelimumab). CIO was used in 27 (9.5%) patients who were counted in both total chemotherapy (274) and any ICI (135). Of the 108 patients treated with prior mono/dual ICI therapy, seven (6.5%) were treated with both a PD-L1 and CTLA-4 inhibitor combination (durvalumab/tremelimumab) while of the 27 treated with prior CIO, four (14.8%) were treated with chemotherapy plus PD-L1 and CTLA-4 inhibitor combination (cisplatin/pemetrexed/durvalumab/tremelimumab). Prior palliative targeted therapy was used in 59 (20.7%) of patients. Median ECOG PS prior to docetaxel was one for ICI mono/dual, CIO and no prior ICI while ECOG PS was ≥2 prior to docetaxel initiation in 18.5%, 11.1% and 18.7% of ICI mono/dual, CIO and no prior ICI, respectively. ECOG PS was unavailable prior to docetaxel initiation for 3.7%, 0% and 15.2% of ICI mono/dual, CIO and no prior ICI, respectively.

The median number of lines of palliative of docetaxel was three (range: 2–6) with 48.4/34.7/16.9% treated in 2L/3L/4L+ setting. The median number of palliative cycles of docetaxel was three (range: 1–28), four (range: 1–44), four (range: 1–18), two (range: 1–11) and seven (range: 1–8) with 2L, 3L, 4L, 5L and 6L, respectively. The main characteristics of the population are summarized in Table 1.

**Table 1:** Characteristics of the study population.

Total Study Population (*n* = 285)		
Age: median (range)	62 (18–85)	
Sex: *n* (%)	M	163 (57.2%)
	F	122 (42.8%)
Cancer Centre: *n* (%)	PMHCC	188 (66.0%)
	TOHCC	48 (16.8%)
	JCC	49 (17.2%)
Smoking status: *n* (%)	Current	58 (21.3%)
	Former	126 (46.3%)
	Never	88 (32.4%)
	Unknown	13
Stage at initial diagnosis: *n* (%)	I	9 (3.2%)
	II	15 (5.3%)
	III	55 (19.3%)
	IV	204 (71.6%)
	Unknown	2
ECOG status at docetaxel initiation: *n* (%)	0	43 (16.7%)
	1	151 (58.5%)
	≥2	64 (24.8%)
	Unknown	27
Histology: *n* (%)	Adenocarcinoma	185 (64.9%)
	Squamous cell	78 (27.4%)
	Large cell	14 (4.9%)
	Neuroendocrine	1 (0.4%)
	Other	6 (2.1%)
PD-L1 expression: *n* (%)	≥50%	37 (19.6%)
	1–49%	49 (25.9%)
	<1%	103 (54.5%)
	Unknown	96
Molecular alterations: *n* (%)	EGFR mutation	47 (20.4%)
	Unknown	55
	KRAS mutation	28 (15.4%)
	Unknown	103
	BRAF mutation	7 (3.9%)
	Unknown	107
	HER2 mutation	6 (3.5%)
	Unknown	114
	ROS1 fusion	4 (2.3%)
	Unknown	108
	ALK rearrangement	3 (1.3%)
	Unknown	58
Metastatic location prior to docetaxel start: *n* (%)	Bone	179 (63.5%)
	Unknown	3
	Pleura	163 (59.5%)
	Unknown	11
	Contralateral lung	147 (54.4%)
	Unknown	15
	Liver	100 (35.5%)
	Unknown	3
	Brain	81 (32.4%)
	Unknown	35
	Adrenal	48 (17.3%)
	Unknown	8
	Skin	18 (6.5%)
	Unknown	7
Prior curative intent therapy: *n* (%)	Total	74 (25.9%)
	Surgery alone	14 (4.9%)
	Surgery + rads	1 (0.4%)
	Surgery + chemo	17 (5.9%)
	Surgery + rads + chemo	8 (2.8%)
	Rads alone	4 (1.4%)
	Rads + chemo	23 (8.1%)
	Rads + chemo + ICI	7 (2.5%)
Total lines of therapy: *n* (%)	2	74 (26.0%)
	3	117 (41.1%)
	4	52 (18.3%)
	5	26 (9.1%)
	6	11 (3.9%)
	7	1 (0.4%)
	8	3 (1.1%)
	9	1 (0.4%)
Docetaxel palliative line of therapy: *n* (%)	2	138 (48.4%)
	3	99 (34.7%)
	4	35 (12.3%)
	5	8 (2.8%)
	6	5 (1.8%)
Prior palliative therapy to docetaxel: *n* (%)	Chemotherapy	274 (96.1%)
	Platinum-based	202 (70.9%)
	Taxane (paclitaxel)	5 (1.8%)
	Any ICI	135 (47.4%)
	ICI mono/dual	108 (38.0%)
	CIO	27 (9.5%)
	Targeted	59 (20.7%)

M—male; F—female; PMHCC—Princess Margaret Cancer Centre; TOHCC—The Ottawa Hospital Cancer Centre; JCC—Juravinski Cancer Centre; ECOG—Eastern Cooperative Oncology Group; EGFR—epidermal growth factor receptor; KRAS—Kirsten rat sarcoma virus; BRAF—B-Raf proto-oncogene, serine/threonine kinase; HER2—human epidermal growth factor receptor 2; ROS1—ROS proto-oncogene 1, receptor tyrosine kinase; ALK—anaplastic lymphoma kinase; rads—radiation therapy; ICI—immune checkpoint inhibitor; CIO—chemo-immunotherapy, mono-monotherapy; BRAF—proto-oncogene, serine/threonine kinase.

### 3.2. Median Overall Survival Based on Patient Characteristics (Table 2)

The mOS for 2L+ docetaxel therapy in the palliative setting was 6.5 months (95% CI: 5.9–7.3). Based on docetaxel line, mOS was 6.4 months, 6.3 months, and 7.2 months for 2L, 3L or 4L+, respectively. Median OS was shorter as performance status deteriorated (8.5 months in ECOG PS—0 patients; 6.8 months in ECOG PS—1 patients; and 4.3 months in ECOG PS—2 patients); that was significant (*p* = 0.0012) comparing ECOG PS 0/1 to ≥2.

Longer OS was seen in KRAS mutant NSCLC patients compared to wildtype (10.2 versus 6.5 months, *p* = 0.0047), and in those without bone metastases at docetaxel initiation (6.8 versus 6.3 months, *p* = 0.048). Among patients previously treated with platinum-based chemotherapy, the median interval from platinum exposure to initiation of second-line or later (2L+) docetaxel was 4.6 months. The mOS was significantly longer in patients with a prolonged interval (≥4.6 months) compared with those with a shorter interval (<4.6 months) since platinum-based chemotherapy (7.0 vs. 4.8 months; *p* = 0.0035).

Notable factors that displayed no impact on mOS with 2L+ docetaxel included any prior ICI treatment compared to no previous ICI therapy (6.6 months vs. 6.3 months; *p* = 0.58), palliative line of docetaxel use (2L, 3L or 4L+), presence of other actionable genomic alterations (EGFR, BRAF, HER2, ROS1, ALK), other metastatic sites (pleura, contralateral lung, brain, adrenal, liver, skin), or 1L palliative therapy used including chemotherapy, ICI, CIO or targeted therapy (6.2 months vs. 6.3 months vs. 8.4 months vs. 6.4 months, respectively; *p* = 0.35). Median OS is reported in Table 2.

**Table 2:** Median overall survival with 2L+ docetaxel in overall population and subgroups.

Patient Group	*n*	mOS (Months; 95% CI)	*p*-Value
Overall	285	6.5 (5.9–7.3)	--------
ECOG PS 0	41	8.5 (3.8–17.2)	0.0012
ECOG PS 1	151	6.8 (6.0–7.9)	
ECOG PS ≥ 2	58	4.3 (3.1–6.2)	
PMHCC	188	6.6 (6.1–7.5)	0.56
TOHCC	48	6.4 (4.7–9.2)	
JCC	49	5.8 (4.6–9.8)	
Any prior ICI treatment	135	6.6 (5.8–8.4)	0.58
No prior ICI treatment	150	6.3 (5.4–7.3)	
Docetaxel 2nd line	138	6.4 (5.3–7.6)	0.84
Docetaxel 3rd line	99	6.3 (4.3–7.8)	
Docetaxel ≥ 4th line	48	7.2 (5.4–9.9)	
Duration since platinum therapy			0.0035
<median (4.6 months)	100	4.8 (3.7–6.4)	
≥median (4.6 months)	102	7.0 (6.0–9.4)	
KRAS mutation present	28	10.2 (3.8–17.2)	0.0047
KRAS mutation absent	154	6.5 (5.7–7.5)	
EGFR mutation present	47	6.1 (3.7–7.5)	0.13
EGFR mutation absent	183	6.5 (5.7–8.1)	
BRAF mutation present	7	2.7 (0.4–29.5)	0.45
BRAF mutation absent	171	6.7 (5.9–7.9)	
HER2 mutation present	6	8.8 (1.3–23.6)	0.88
HER2 mutation absent	165	6.5 (5.7–7.5)	
ROS1 fusion present	4	8.3 (0.4–33.3)	0.66
ROS1 fusion absent	173	6.5 (5.8–7.5)	
ALK rearrangement present	3	5.9 (0.4–6.0)	0.10
ALK rearrangement absent	224	6.5 (5.9–7.9)	
Bone metastasis	179	6.3 (4.8–7.1)	0.048
No bone metastasis	103	6.8 (5.9–8.5)	
Brain metastasis	81	6.0 (4.3–7.2)	0.069
No brain metastasis	169	6.6 (6.1–8.1)	
Pleural metastasis	163	6.3 (5.4–6.9)	0.90
No pleural metastasis	111	7.5 (5.8–8.5)	
Contralateral lung metastasis	147	6.3 (5.2–7.2)	0.52
Non contralateral lung metastasis	123	6.9 (5.9–8.2)	
Liver metastasis	100	6.1 (4.7–6.9)	0.090
No liver metastasis	182	7.0 (6.0–8.2)	
Adrenal metastasis	48	3.7 (3.0–5.2)	0.10
No adrenal metastasis	229	6.9 (6.3–7.9)	
Skin metastasis	18	4.7 (1.7–8.4)	0.46
No skin metastasis	260	6.5 (6.0–7.5)	
1L chemotherapy	199	6.2 (5.2–8.1)	0.35
1L ICI mono/dual therapy	21	6.3 (3.0–9.2)	
1L CIO	22	8.4 (2.3–14.6)	
1L targeted therapy	43	6.4 (5.4–8.1)	

ECOG—Eastern Cooperative Oncology Group; PMHCC—Princess Margaret Cancer Centre; TOHCC—The Ottawa Hospital Cancer Centre; JCC—Juravinski Cancer Centre; EGFR—epidermal growth factor receptor; KRAS—Kirsten rat sarcoma virus; HER2—human epidermal growth factor receptor 2; BRAF—B-Raf proto-oncogene, serine/threonine kinase; ROS1—ROS proto-oncogene 1, receptor tyrosine kinase; ALK—anaplastic lymphoma kinase; ICI—immune checkpoint inhibitor; CIO—chemo-immunotherapy.

### 3.3. Response Endpoints with 2L+ Docetaxel

**Figure 1:**
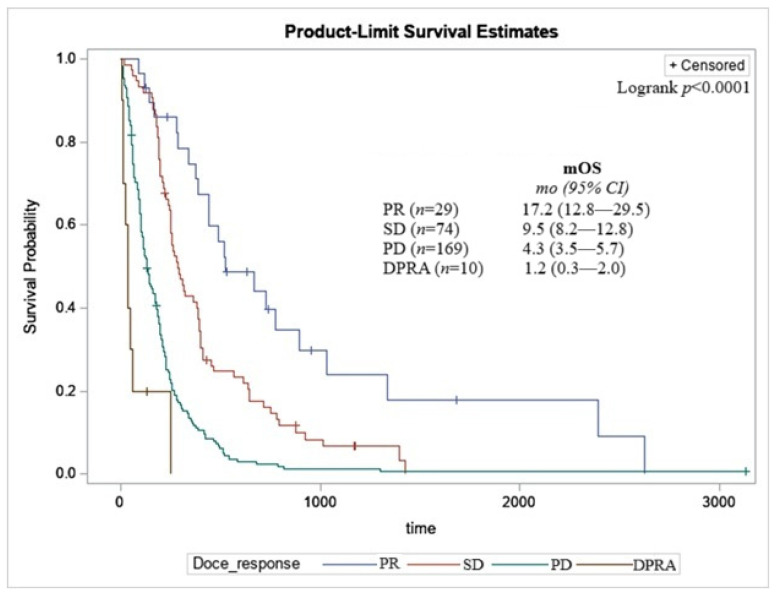
Kaplan–Meier curve of OS based on 2L+ docetaxel response. PR—partial response, SD—stable disease, PD—progressive disease, DPRA—death prior to response assessment. Time reported in days since docetaxel start until death from any cause.

Based on investigator-assessed response to 2L+ docetaxel use on chart review, 10.3% of patients had a partial response (PR), 26.2% had stable disease (SD), 59.9% had progressive disease (PD) and 3.5% dying prior to response assessment (DPRA; Table 3). mOS with palliative docetaxel based on disease response was 17.2 months (95% CI: 12.8–29.5) for patients assessed to have a PR, 9.5 months (95% CI: 8.2–12.8) for SD, 4.3 months (95% CI: 3.5–5.7) for PD and 1.2 months (95% CI: 0.3–2.0) for DPRA (logrank *p* < 0.0001; Table 3 and Figure 1).

### 3.4. Univariate Analysis with Hazard Ratios Based on Patient Characteristics (Table 4)

On univariate analysis, factors associated with an increased hazard ratio of death included patients with an ECOG PS of one (HR 1.82; 95% CI: 1.23–2.69; *p* = 0.0026) or two (HR 2.41; 95% CI: 1.55–3.76; *p* < 0.0001) compared to an ECOG PS of 0 at the start of 2L+ palliative docetaxel (Table 4). Other factors associated with an increased hazard of death with 2L+ docetaxel included large cell histology (HR 1.74; 95% CI: 1.01–3.03; *p* = 0.046) compared to adenocarcinoma histology, duration of previous line to docetaxel ≤6 months (HR 1.40; 95% CI 1.03–1.90; *p* = 0.031) compared to >6 months and prior palliative vinorelbine (HR 2.64; 95% CI: 1.03–6.77; *p* = 0.043) compared to prior palliative gemcitabine (Table 4). Factors associated with a decreased hazard of death included tumours that possessed a KRAS mutation (HR 0.53; 95% CI: 0.34–0.83; *p* = 0.0054) and never having smoked (HR 0.73; 95% CI: 0.55–0.98; *p* = 0.033) compared to former smokers. Notable characteristics which did not show a difference in univariate hazard ratio analysis included prior treatment (ICI mono/dual therapy, CIO or targeted therapy), selected molecular alterations (EGFR, BRAF, HER2, ROS1, ALK), PD-L1 status or prior curative intent treatment.

**Table 4:** Univariate and multivariate analysis with HRs between different prognostic factors associated with hazard of death treated with 2L+ docetaxel.

	Univariate Analysis		Multivariate Analysis	
Patient Factor	HR (95% CI)	*p*-Value	HR (95% CI)	*p*-Value
Sex (M vs. F)	1.01 (0.79–1.28)	0.99		
Age (increase 1 year)	1.01 (0.99–1.01)	0.87		
Smoking Status (former vs. never)	0.73 (0.55–0.98)	0.033	0.61 (0.34–1.07)	0.085
Smoking Status (current vs. never)	0.85 (0.60–1.22)	0.35		
ECOG (1 vs. 0)	1.82 (1.23–2.69)	0.0026	2.26 (1.15–4.43)	0.018
ECOG (≥2 vs. 0)	2.41 (1.55–3.76)	<0.0001	2.62 (1.27–5.41)	0.0091
Site (JCC vs. TOHCC)	1.07 (0.70–1.65)	0.76		
Site (PMHCC vs. TOHCC)	1.18 (0.85–1.65)	0.32		
Histology (SCC vs. adeno)	1.26 (0.96–1.66)	0.10		
Histology (large cell vs. adeno)	1.74 (1.01–3.01)	0.046	2.15 (0.99–4.66)	0.051
PD-L1 (1–49% vs. ≥50%)	0.88 (0.57–1.36)	0.56		
PD-L1 (<1% vs. ≥50%)	1.22 (0.84–1.80)	0.30		
KRAS (mutated vs. non-mutated)	0.53 (0.34–0.83)	0.0054	0.59 (0.37–0.94)	0.026
EGFR (mutated vs. non-mutated)	1.29 (0.93–1.78)	0.13		
BRAF (mutated vs. non-mutated)	0.73 (0.32–1.66)	0.45		
HER2 (mutated vs. non-mutated)	0.94 (0.42–2.12)	0.88		
ROS1 (fusion vs. non-fusion)	0.80 (0.30–2.16)	0.66		
ALK (rearrangement vs. non-rearrangement)	2.53 (0.80–7.99)	0.11		
Prior ICI mono/dual therapy (yes vs. no)	0.98 (0.77–1.25)	0.87		
Prior CIO (yes vs. no)	1.07 (0.70–1.62)	0.76		
Prior targeted therapy (yes vs. no)	1.11 (0.83–1.49)	0.48		
Duration of previous line of therapy (≤6 m vs. >6 m)	1.40 (1.03–1.90)	0.031	1.71 (0.95–3.07)	0.072
Prior curative intent immunotherapy	1.53 (0.72–3.26)	0.27		
Prior curative intent chemo-immunotherapy	1.07 (0.70–1.61)	0.76		
Prior curative intent targeted therapy	1.11 (0.83–1.49)	0.48		
Prior curative intent chemotherapy	1.18 (0.88–1.59)	0.27		
Prior curative intent radiation	1.37 (0.98–1.90)	0.062		
Prior curative intent surgery	1.04 (0.74–1.45)	0.84		

M—male; F—female; ECOG—Eastern Cooperative Oncology Group; PMHCC—Princess Margaret Cancer Centre; TOHCC—The Ottawa Hospital Cancer Centre; JCC—Juravinski Cancer Centre; SCC—squamous cell carcinoma; Adeno—adenocarcinoma; PD-L1—programmed death ligand 1; EGFR—epidermal growth factor receptor; KRAS—Kirsten rat sarcoma virus; BRAF—B-Raf proto-oncogene, serine/threonine kinase; HER2—human epidermal growth factor receptor 2; ROS1—ROS proto-oncogene 1, receptor tyrosine kinase; ALK—anaplastic lymphoma kinase; ICI—immune checkpoint inhibitor; CIO—chemo-immunotherapy.

### 3.5. Multivariate Analysis with Hazard Ratios Based on Patient Characteristics (Table 4)

On multivariate analysis, the presence of a KRAS mutation in a tumour was independently associated with improved survival (HR 0.59; 95% CI: 0.37–0.94; *p* = 0.026) compared to KRAS wild type. An ECOG PS at time of 2L+ initiation of palliative docetaxel of either one (HR 2.26; 95% CI: 1.15–4.43; *p* = 0.018) or two (HR 2.62; 95% CI: 1.27–5.41; *p* = 0.0091) was associated with a more than twofold increase in risk of death compared to ECOG PS 0. Other characteristics which showed a predictive difference on univariate analysis including former vs. never smoker, large cell vs. adenocarcinoma histology, duration of previous line to docetaxel ≤6 months vs. >6 months and prior palliative vinorelbine compared to gemcitabine were all found to not be predictive on multivariate analysis.

## 4. Discussion

In this multicenter retrospective study, we report the real-world evidence (RWE) of palliative docetaxel monotherapy in the 2L+ setting in patients with advanced NSCLC upon progression after 1L chemotherapy, ICI, CIO, or targeted therapy. We also report on specific patient characteristics and predictors of response that affect mOS differences and hazard of death to 2L+ docetaxel therapy. Our findings suggest that docetaxel remains a clinically relevant option in the post-platinum setting, even in the era of targeted therapies and immune checkpoint inhibitors.

The mOS of our overall cohort was 6.5 months (95% CI: 5.9–7.3 months), which is consistent with outcomes reported in pivotal trials such as TAX 317 (7.5 months) which helped establish docetaxel as the standard of care in 2L treatment of NSCLC [7]. Previously reported 2L salvage docetaxel has been shown to have a mOS of 6.4–9.7, months which is consistent with our study [8,9,16,17,19,25,26,27]. Our RWE mOS was on the lower end compared to previous studies. One explanation could be that in our study 25% of patients were ECOG PS ≥ 2, whereas several trials which reported the highest mOS did not include patients with ECOG PS ≥ 2 [16,17,20,21,22,23]. This is standard for most clinical trials but does not fit with everyday patients in the clinic. Our mOS is consistent with the initial TAX study results, in which patients with ECOG performance status 2 comprised 24% of the trial population, comparable to the 25% observed in our cohort [7]. Although the absolute survival benefit is modest, it is important to recognize the role of docetaxel in heavily pretreated patients, particularly those who have progressed on ICI, CIO, or targeted agents. Our data suggests that despite the addition of patients previously treated with immunotherapy (ICI mono/dual therapy, CIO) or targeted agents, overall treatment population mOS aligns with previous OS data in an era without immunotherapy. Our data continues to support the role of docetaxel use in the 2L+ setting in patients with metastatic NSCLC after progression, regardless of prior therapy.

One of the earliest clinical trials examining docetaxel as any line agent after ICI in clinical practice was Tamura et al. who looked at docetaxel with or without ramucirumab after nivolumab [28]. They reported better response to treatment post-ICI with improved ORR (27.8% vs. 16.0%). The group also reported that 2L taxane monotherapy had a mOS of 6.4 months (95% CI: 5.0–12.9) while taxane plus anti-angiogenic achieved the longest 2L mOS—not reached (95% CI: 5.8-NR). Previous studies have also shown a possible enhanced effect of 2L docetaxel post-progression on ICI with the addition of ramucirumab (8.6 vs. 19.8 months) but no difference without prior ICI therapy [27]. In the United States, ramucirumab is a treatment option with 2L docetaxel, while in Canada this combination is not funded, with docetaxel being the most used treatment option after progression on ICI, CIO or targeted therapy.

Our study population was reflective of routine clinical practice, with a heterogeneous mix of histologies, ECOG PS, total lines of therapy, line of docetaxel use and lines of therapy prior to docetaxel. A substantial proportion of patients received docetaxel as third-line or later, emphasizing its utility in salvage settings. Our driver mutation profile was similar to previous studies in Western populations with notably lower numbers of KRAS mutant tumours (15.4% in our study vs. 30–40%) and higher rates of EGFR mutations (20.4% in our study vs. 10–16%) in North American populations [29]. A total of 103 (36.1%) patient tumours had not been evaluated for KRAS mutation due to a portion of our cohort existing in a time where KRAS testing was not the standard of care. Of the 182 who had their tumours evaluated, 28 were positive (15.4%). Despite this, our rates of KRAS mutation were lower than expected. Possibly explained by a subset of patients with SCC histology (27.4%), which are known to display lower rates of KRAS alterations compared to adenocarcinomas with most SCC histology not being evaluated based on standard of care practices in Ontario, Canada at that time. Only one patient with a SCC histology had a KRAS mutant tumour while of the adenocarcinoma patients who had available KRAS testing, 27/122 (22.1%) had a KRAS mutation. Slightly higher rates than expected of EGFR mutant tumours could be explained by a high proportion of patients treated with prior targeted therapy (78.7%) and never smokers (70.2%), selecting for fitter patients more likely to be treated with later lines of therapy. We also noted slightly higher rates of PD-L1 negative (<1%) at 54.5%. Although a recent global multicentre observational study reviewed real-world prevalence data and found that among 2435 patients 48.4% had a PD-L1 < 1% while 22.2% were PD-L1 high (≥50%), which was similar to our data at 19.6% [30]. Variability in PD-L1 status has been shown be due to the specific assay clone used (22C3, 28-8, SP263), inter-laboratory variability, ethnic differences and tumour selection of prior therapy [31].

Patient characteristics demonstrating predictive value in increasing mOS with 2L+ docetaxel included ECOG PS 0/1, duration since platinum therapy ≥ median duration since exposure to platinum use (4.6 months), absence of bony metastatic disease or patients with tumours possessing a KRAS mutation. Not surprisingly, patients who had a better functional status (ECOG PS 0/1) prior to the start of 2L+ docetaxel had improved OS. Conversely, having a higher ECOG PS of ≥2 was associated with a 141% increased hazard of death on univariate analysis and a sustained 162% increased hazard of death on multivariate analysis compared to patients with an ECOG PS 0 at time of docetaxel initiation. Duration since platinum therapy as a predictor is supported by previous studies where patients that progressed on 1L CIO who were considered responders (1L PFS ≥ 6 months) had improved OS compared to those who were considered resistant (PFS < 6 months) [19]. This implies that patients who progressed within a shorter interval after discontinuing platinum-based therapy (<median time since treatment) had worse overall survival with docetaxel than those with a longer post-platinum treatment interval. The absence of bone metastasis improving mOS with palliative docetaxel is hypothesized in the literature with potential mechanisms including poor drug biodistribution in bone [32], bone microenvironment providing a sanctuary site via the CXCL12-CXCR4 axis [33] and blunting of docetaxel efficacy with tumour–stroma signalling dependent on RANK/RANKL/OPG axis [34].

Histology subtype also showed a predictive characteristic with large cell lung cancers displaying a 74% increased hazard of death compared to adenocarcinomas on univariate analysis. This is supported in the literature as large cell lung cancers have been consistently associated with a worse OS compared to adenocarcinoma; although, no previous studies have associated them with response to palliative docetaxel [35,36]. It should be noted that the number of patients were low in the large cell histology (14; 4.9%) and, therefore, this predictive characteristic should be viewed more as hypothesis-generating and something for future studies to explore. Patients with duration of prior therapy ≤ 6 months had an increased hazard of death on univariate analysis (HR 1.4, *p* = 0.031) and a similar trend on multivariate analysis (HR 1.71, *p* = 0.072). These findings are consistent with prior literature which has shown that patients who were considered resistant to 1L treatment (progression < 6 months) or prior ICI-based treatment lasting ≥6 months had superior outcomes with OS and PFS [19,37]. The only two patient characteristics which continued to display an association on multivariate analysis included ECOG PS 1 vs. 0/≥2 vs. 0 and tumours possessing a KRAS mutation with treatment of 2L+ docetaxel in metastatic NSCLC. This is consistent with prior literature demonstrating poorer outcomes with second-line docetaxel in patients with ECOG PS 2 compared with alternative chemotherapies such as pemetrexed [8]. Based on these results, it would be reasonable to consider alternative treatment options or best supportive care for patients who have a poor ECOG PS (≥2) given the noted increased hazard of death with 2L+ docetaxel. Lastly, site-specific mOS demonstrates no significant differences across participating centres (Table 2). This suggests that inter-site variation in local treatment patterns, radiologic review, and imaging schedules did not materially influence endpoint assessment or introduce measurable centre-level confounding in this cohort.

Longer mOS benefit and improved HR (47% reduction in the hazard of death) were shown in patients’ tumours that had a KRAS mutation. Possessing a KRAS mutation in metastatic NSCLC is thought to carry a worse OS compared to KRAS wild-type while the use of ICIs and PD-L1 status have been shown to increase OS with KRAS mutant tumours [38]. In our study, analysis of all patients with KRAS mutant tumours revealed, 46% were treated with prior ICI while 29%, 14% and 11% were treated with prior chemotherapy, targeted therapy, or CIO, respectively. PD-L1 high (≥50%) was present in only 7% of these patients while 57% and 25% were PD-L1 1–49% and <1%, respectively. Therefore, the mOS benefit and decreased hazard of death can not be fully attributed in our patient population to prior treatment with ICI or PD-L1 high status. Further analysis of KRAS mutation subtypes showed a higher-than-expected proportion of G12A (46.4%) compared to lower rates in real-world databases (9–10%) and lower than expected G12C variants (25.0%) compared to previous studies (39–49%) [39,40]. Although these differences were present, KRAS mutation subtype is not associated with survival difference in the current era of immunotherapy [39]. Thus, despite our rates of KRAS subtype mutation being slightly different from large KRAS databases, we would not expect this increased rate of KRAS G12A to play a role on our protective effect noted with KRAS mutation status with 2L+ docetaxel therapy. Characteristics which displayed no predictive OS benefit or HR effect on survival with 2L+ docetaxel included prior therapy (chemo, ICI, CIO, targeted), palliative line of docetaxel, EGFR alterations, or other sites of metastasis aside from bone (contralateral lung, pleura, liver, brain). Exploratory analyses demonstrated no difference in ECOG performance status prior to docetaxel initiation between patients with and without prior ICI exposure, including median ECOG PS (one in both groups) and the proportion with ECOG PS ≥2, suggesting no baseline performance status imbalance between groups and further supporting our findings. The lack of impact on mOS of certain actionable genomic alterations should be viewed with caution and can not be used to draw any conclusions around predictive factors for response to 2L+ docetaxel due to the low number of patients with HER2 (n = 6; 3.5%), BRAF (n = 7; 3.9%), ROS1 (n = 4; 2.3%) and ALK (n = 3; 1.3%) alterations. These findings support a more nuanced patient selection for docetaxel therapy, although the retrospective design precludes definitive conclusions.

Our response rates (ORR: 10.3%; DCR: 36.5%) were similar to previously published data. Previous studies of docetaxel in the 2L setting in NSCLC have noted ORR in the range of 8–21% and DCR in the range of 48–63% [6,18,41,42]. One explanation for why our RWE data shows a lower end of response could be related to the overall lower ECOG PS used in these studies (ECOG PS 0–1) compared to our study cohort. The patient characteristic which displayed the greatest mOS benefit was investigator-assessed best response of a PR (17.2 months; 95% CI: 12.8–29.5; Figure 1), which was 1.7-fold higher than the next predictor of KRAS mutation. Highlighting the importance of clinician assessment during treatment, which we demonstrate can be closely associated with mOS benefit.

As a real-world, retrospective analysis, this study is subject to several limitations, including potential selection bias and incomplete data capture. Heterogeneity in practice patterns across centres, variability in supportive care, and the absence of centralized radiologic review may have influenced outcomes. As three independent academic institutions were involved, no centralized molecular review was in place during the study period with molecular analysis conducted at local laboratories using site-specific validated assays. Consequently, there was variability in detection platforms, reagents, and interpretation criteria, particularly in the pre-NGS era. Because a portion of the study period occurred prior to 2015, when ICI/CIO use was largely restricted to clinical trial settings and monotherapy became standard practice subsequently, the observed proportion of patients with prior immunotherapy exposure may have been influenced. Incomplete molecular and PD-L1 data limited subgroup analyses. All patients in this study were treated at comprehensive cancer centres, which may limit the generalizability to community practice where patient populations and access to therapies and clinical trials may differ. Treatment-related adverse events were not captured, precluding assessment of toxicity and real-world management. Reason for docetaxel discontinuation or switch was not captured and thus limits assessment of real-world treatment patterns. Mortality data were derived solely from medical records without external linkage, potentially leading to survival overestimation, and cause-specific mortality could not be assessed. Despite these limitations, this study represents the first real-world evaluation of docetaxel monotherapy efficacy and predictors of response in a diverse, advanced NSCLC population treated in the 2L+ setting.

Future studies should evaluate rational combination strategies to enhance docetaxel efficacy, including antiangiogenic agents and emerging immunomodulators, particularly in ICI-pretreated patients. As antibody–drug conjugates increasingly move into earlier lines of therapy, docetaxel may be further relegated to later-line settings, underscoring the need to better define patient characteristics associated with benefit. Incorporation of patient-reported outcomes will also be critical to contextualizing clinical benefit and quality of life in the refractory setting. As treatment paradigms evolve, clarifying the optimal role of docetaxel will remain essential to individualized care beyond immunotherapy.

## 5. Conclusions

To our knowledge, this is the largest multi-institute real-world study that has systematically investigated the patient characteristics and prognostic factors affecting OS with 2L+ docetaxel in patients treated with prior ICI, CIO, or targeted therapy. In this RWE study, docetaxel use in 2L+ setting showed similar mOS to initial clinical trials, regardless of prior ICI use. We show that prior use of palliative ICI, CIO or targeted therapy had no effect on OS with 2L+ docetaxel. Factors which were associated with prolonged OS with docetaxel as an independent predictor on multivariate analysis included presence of a KRAS mutation in tumours and a good ECOG PS (0/1) prior to docetaxel initiation. Our real-world data supports the overwhelming body of clinical trial evidence that continues to demonstrate a modest level of efficacy for single-agent docetaxel in the 2L+ setting. With the therapeutic landscape for advanced NSCLC evolving significantly with the advent of ICIs and targeted agents, we show there remains a critical need for effective cytotoxic options in patients who exhaust these modalities. Docetaxel, especially in monotherapy, continues to be a pragmatic and accessible option.

## Figures and Tables

**Table 3:** Response endpoints in 2L+ docetaxel.

Response	*n* (%)	Median OS (Months; 95% CI)	*p*-Value
CR	0	------	<0.0001
PR	29 (10.3%)	17.2 (12.8–29.5)	
SD	74 (26.2%)	9.5 (8.2–12.8)	
PD	169 (59.9%)	4.3 (3.5–5.7)	
DPRA	10 (3.5%)	1.2 (0.3–2.0)	
Unknown	3	------	
ORR (CR + PR)	29 (10.3%)		
DCR (CR + PR + SD)	103 (36.5%)		

CR—complete response; PR—partial response; SD—stable disease; PD—progressive disease; DPRA—death prior to response assessment; ORR—overall response rate; DCR—disease control rate.

## Data Availability

The data presented in this study is available on request from the corresponding author due to ethical and privacy restrictions related to patient confidentiality and institutional/REB data-sharing policies.

## Abbreviations

The following abbreviations are used in this manuscript:

NSCLC	Non-small cell lung cancer
SCC	Squamous cell carcinoma
mOS	Median overall survival
ICI	Immune checkpoint inhibitor
CIO	Chemoimmunotherapy
ECOG PS	Eastern Cooperation Oncology Group performance status
ORR	Overall response rate
DCR	Disease control rate
2L+	Second-line or later
PR	Partial response
SD	Stable disease
PD	Progressive disease
DPRA	Dying prior to response assessment
RWE	Real-world evidence
